# Deep Learning Models for Segmenting Non-perfusion Area of Color Fundus Photographs in Patients With Branch Retinal Vein Occlusion

**DOI:** 10.3389/fmed.2022.794045

**Published:** 2022-06-30

**Authors:** Jinxin Miao, Jiale Yu, Wenjun Zou, Na Su, Zongyi Peng, Xinjing Wu, Junlong Huang, Yuan Fang, Songtao Yuan, Ping Xie, Kun Huang, Qiang Chen, Zizhong Hu, Qinghuai Liu

**Affiliations:** ^1^Department of Ophthalmology, The First Affiliated Hospital of Nanjing Medical University, Nanjing, China; ^2^School of Computer Science and Engineering, Nanjing University of Science & Technology, Nanjing, China; ^3^Department of Ophthalmology, The Affiliated Wuxi No.2 People's Hospital of Nanjing Medical University, Wuxi, China; ^4^The First School of Clinical Medicine, Nanjing Medical University, Nanjing, China

**Keywords:** deep learning, non-perfusion area, color fundus photograph, branch retinal vein occlusion, artificial intelligence, automatic segmentation

## Abstract

**Purpose:**

To develop artificial intelligence (AI)-based deep learning (DL) models for automatically detecting the ischemia type and the non-perfusion area (NPA) from color fundus photographs (CFPs) of patients with branch retinal vein occlusion (BRVO).

**Methods:**

This was a retrospective analysis of 274 CFPs from patients diagnosed with BRVO. All DL models were trained using a deep convolutional neural network (CNN) based on 45 degree CFPs covering the fovea and the optic disk. We first trained a DL algorithm to identify BRVO patients with or without the necessity of retinal photocoagulation from 219 CFPs and validated the algorithm on 55 CFPs. Next, we trained another DL algorithm to segment NPA from 104 CFPs and validated it on 29 CFPs, in which the NPA was manually delineated by 3 experienced ophthalmologists according to fundus fluorescein angiography. Both DL models have been cross-validated 5-fold. The recall, precision, accuracy, and area under the curve (AUC) were used to evaluate the DL models in comparison with three types of independent ophthalmologists of different seniority.

**Results:**

In the first DL model, the recall, precision, accuracy, and area under the curve (AUC) were 0.75 ± 0.08, 0.80 ± 0.07, 0.79 ± 0.02, and 0.82 ± 0.03, respectively, for predicting the necessity of laser photocoagulation for BRVO CFPs. The second DL model was able to segment NPA in CFPs of BRVO with an AUC of 0.96 ± 0.02. The recall, precision, and accuracy for segmenting NPA was 0.74 ± 0.05, 0.87 ± 0.02, and 0.89 ± 0.02, respectively. The performance of the second DL model was nearly comparable with the senior doctors and significantly better than the residents.

**Conclusion:**

These results indicate that the DL models can directly identify and segment retinal NPA from the CFPs of patients with BRVO, which can further guide laser photocoagulation. Further research is needed to identify NPA of the peripheral retina in BRVO, or other diseases, such as diabetic retinopathy.

## Introduction

Retinal non-perfusion area (NPA) is a vision-threatening condition that is strongly associated with retinal neovascularization (NV) (1), vitreous hemorrhage, and macular edema in retinal diseases, such as diabetic retinopathy (DR) (2) and branch retinal vein occlusion (BRVO) (3, 4). In ophthalmological practice, a standard and early location of NPA is critical for clinical decision-making, such as prompt laser photocoagulation (2, 5).

It is acknowledged that fundus fluorescein angiography (FFA) is the gold standard for circling the NPA. However, FFA might not be acceptable for those with serious liver or kidney dysfunction, or with a drug allergy history (6). Serious complications can occur with FFA, such as an anaphylactic reaction (7). In other circumstances, when patients with undetermined vitreous hemorrhage are receiving diagnostic pars plana vitrectomy, laser photocoagulation is usually performed after the clearance of vitreous hemorrhage, which might not be precise if in the absence of FFA.

Recently, artificial intelligence (AI) has been showing promising potential in assisting the diagnosis and treatment of medical conditions based on medical imaging (8). Deep learning (DL), as a particular form of AI, allows systems to learn predictive features from raw images based on a large dataset of labeled examples without specifying rules or features. In ophthalmology, a number of studies have demonstrated the feasibility of using DL algorithms for the identification of various retinal diseases, such as diabetic retinopathy (DR) (9). In the study of Arcadu F et al. (10), DL was reported to be able to predict key quantitative traditional optical coherence tomography (TD-OCT) measurements related to macular thickening from color fundus photographs (CFPs) and enhance the efficiency of diabetic macular edema (DME) diagnosis in teleophthalmology programs. In a more recent investigation, DL was used to automatically segment the NPA in OCTA images of patients with DR (3, 10), which provides a measure for monitoring peripheral vascular perfusion in eyes with early diabetic disease and the therapeutic response of eyes undergoing treatment for proliferative diabetic retinopathy (PDR) or DME (11–13). In the present study, we stepped forward and employed AI to interpret NPA areas directly from CFP images.

Branch retinal vein occlusion refers to the obstruction of a branch of the retinal vein at an arteriovenous crossing, which has become the second most common retinal vascular disease after diabetic retinopathy. Studies have found the risk of BRVO to be 1.6–1.8%. The compression of the vein is thought to cause turbulent blood flow that leads to thrombus formation, and the thrombosis can result in engorged veins frequently accompanied by variable amounts of retinal non-perfusion. Branch retinal vein occlusion may present with a sudden onset of painless vision loss or visual field defect correlating to the area of perfusion of the obstructed vessels. The complete dilated fundus examination can help diagnose early BRVO (14, 15). As reported, retinal NV developed in 9% within 12 months from onset and in 15% within 36 months from onset, and optic disc NV developed in 8.3% within 12 months from onset and in 10.4% within 30 months from onset (16). Thus, scatter argon laser photocoagulation is promising for use in the involved sector in major BRVO, especially when NV exists (17). Given that BRVO is relatively uniform in clinic and is characterized by blockage of the branch retinal vein and non-perfusion status where laser therapy is needed, here, we first tried the DL methods in BRVO (18).

## Methods

### Dataset

The study was conducted in accordance with the Declaration of Helsinki, and the study protocol was approved by the Ethics Committee of the First Affiliated Hospital of Nanjing Medical University (2021-SR-330).

The dataset used in this study included CFPs and FFA images from patients diagnosed with BRVO from March 2018 to October 2020 in the First Affiliated Hospital of Nanjing Medical University. CFPs were captured with a digital retinal camera (Canon INC, CR-2 AF, Japan) and FFA was performed with the Spectralis HRA2 (Heidelberg Engineering, Heidelberg, Germany). The inclusion criteria were as follows: (1) patients with the diagnosis of BRVO after FFA examination. (2) patients with simultaneous CFPs and FFA images; and (3) high quality CFPs and FFA images. The fair-quality images were defined as photographs with partial visibility of distinct retinal vessels, optic nerve, and retinal backgrounds. Photographs with blurred retinal components that abnormal lesions could not be distinguished were defined as poor-quality images. We used Matlab software (MATLAB 8.3 R2014a, The MathWorks, Natick, Massachusetts) to manually register the CFPs and FFA images and then scaled them to the size of 1,024 pixels × 1,024 pixels.

### Research Strategies

**Figure 1:**
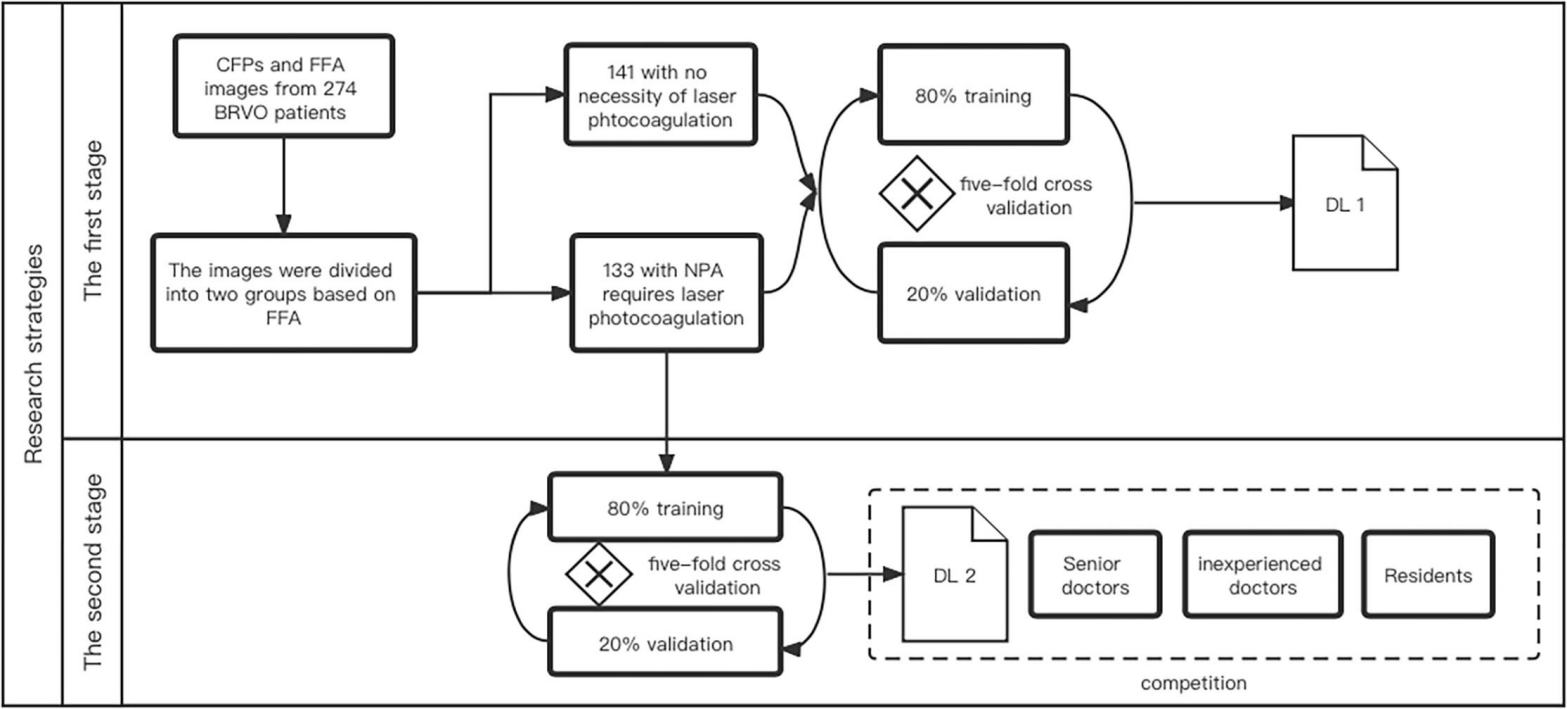
The research strategy of this study.

**Figure 2:**
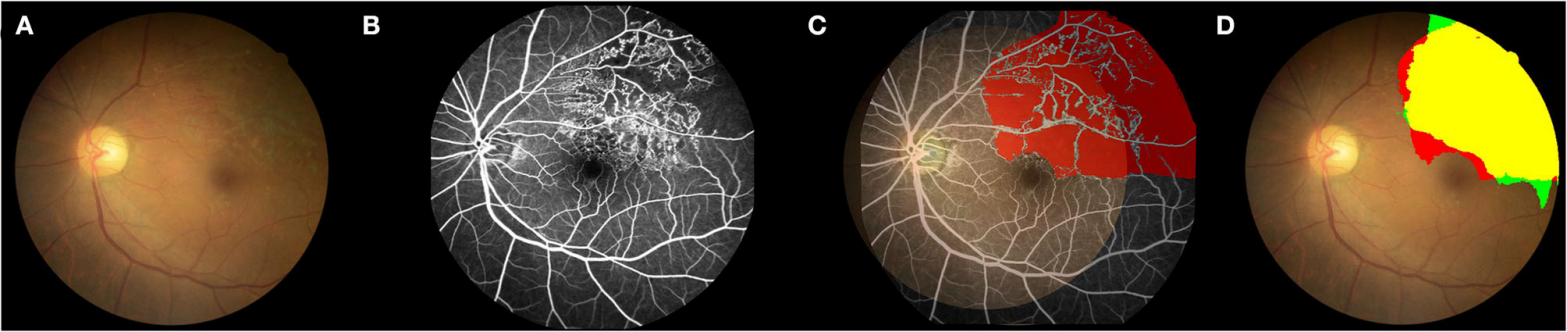
Example of the comparison between the second DL model and FFA in detecting the non-perfusion area. **(A)** Color fundus photograph (CFP). **(B)** FFA image shows the superior-temporal non-perfusion area. **(C)** Labeling the NPA in the CFP according to the FFA images. The CFP image was registered with the corresponding FFA image through the superposition of the blood vessels. The NPA in the FFA image was then labeled in the CFP for the following DL training. **(D)** TP, True positive; yellow region; TN, true negative, colorless region; FP, false positive, green region; and FN, false negative, red region. Green plus yellow shows the NPA indicated by the DL model; red plus yellow shows the true NPA indicated by FFA. DL, deep learning; CFP, color fundus photograph; FFA, fundus fluorescein angiography; NPA, non-perfusion area.

Figure 1 shows the main research strategies of the work. The CFPs and FFA images of diagnosed BRVO cases were reviewed by three experienced ophthalmologists. In the first step, the BRVO cases were divided into two groups, with or without the need for prompt laser photocoagulation. In the former cases, scatter laser therapy was performed on the involved sector of BRVO and was done only if there were neovascular vessels. The latter cases were defined as those with fresh retinal hemorrhage, low-quality CFPs, and FFA images, or with no NPA. The first DL model was trained to differentiate the two BRVO statuses. Next, we removed the CFPs and FFA images with no need for prompt laser photocoagulation. We then registered the CFP images with the FFA through the superposition of the blood vessels. The NPA in the FFA images were then labeled in the CFP for the second DL training (Figure 2). Finally, the segmentation ability of the second DL model is compared with that of three doctors.

### Deep Learning Algorithms

To automatically figure out the status of BRVO in the first step, we employed a convolutional neural network (CNN) with a (VGG)^1^ (19) architecture, which was typically designed for the image classification tasks. To automatically segment out the NPA regions in the second step, we employed another CNN with a modified U-Net (20) architecture. The U-Net architectures were widely used in the medical image segmentation tasks (21, 22).

Both two DL models are trained in an end-to-end manner, which means that we can get the classification and the segmentation results without any subsequent processing.

### Network Architecture

We used a VGG network consisting of 13 convolutional layers, 3 fully connected layers, and one extra adaptive averaging pooling layer between convolutional layers and fully connected layers. The first 13 convolutional layers extract sematic features from CFPs from 3 channels to 512 channels and process 5 times max-pooling as spatial downsampling. For enhancing the feature extraction ability, we used per-trained parameters in ImageNet (23) as the initial parameters of the convolutional layers. After extracting the features of 512 dimensions by the last convolutional layer, the adaptive averaging pooling layer was used to resize the special size of feature to 1 × 1, which led to the feature being a vector of 512 dimension. Finally, three fully connected layers map the features from 512 to 4,096, from 4,096 to 4,096, and from 4,096 to 2, respectively. Thus, we get the probabilities of two states of the BRVO.

**Figure 3:**
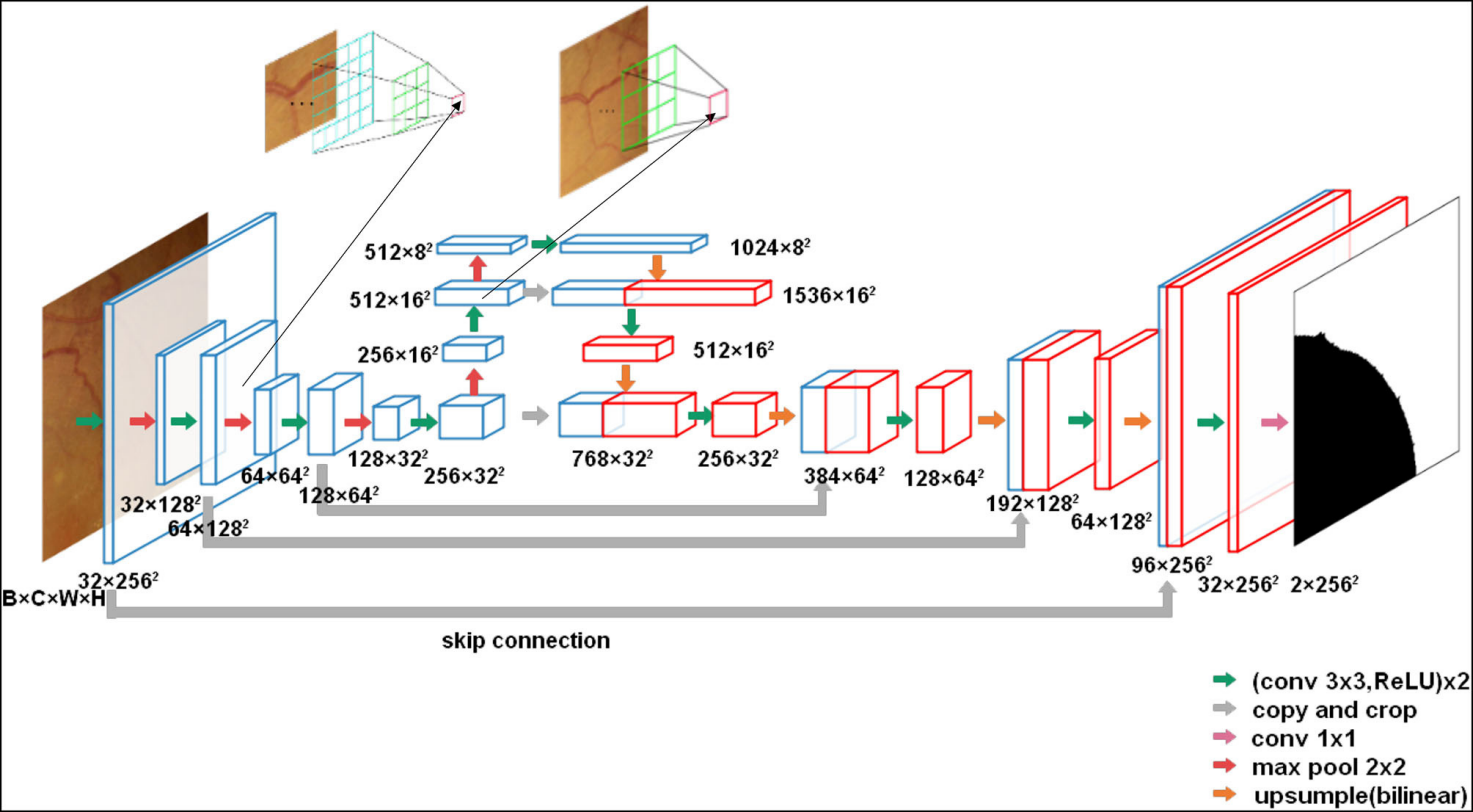
The U-net architecture (3 × 256 × 256 input, for instance). Each green arrow corresponds to two 3 × 3 convolutions each followed by a rectified linear unit (ReLU). Red arrows correspond to a 2 × 2 max pooling operation with stride 2 for downsampling. The orange arrows indicate upsampling with bilinear interpolation. The gray arrows indicate the skip-connection in the U-Net. A 1 × 1 convolution is represented by a purple arrow at the end of the model.

The U-Net network we used is an encoder-decoder architecture with skip-connections to concatenate features with the same resolution of the encoder and the decoder. Thus, it can retain pixel-level detail information at different resolutions, which is important for pixel-wise segmentation. The detail of the network is shown in Figure 3.

In comparison with the original U-Net architecture, we increased the network depth to achieve a large enough receptive field. Meanwhile, to reduce the parameters, the number of convolutional filters was increased from 32 to 1,024 in the encoder. Moreover, to obtain the same output size as the input CFPs, we used a 3 × 3 convolution kernel size with padding 1. In total, the network has 23 convolutional layers.

### Data Augmentation

Supervised learning with neural networks usually requires a large number of image-label pairs. The data of BRVO were relatively small for training DL models and were hard to expand because of the strict inclusion criteria. To mitigate the data requirements of DL models, we used several data augmentation strategies.

We randomly rotated, resized, and flipped the CFPs for both tasks, which can teach models about rotation invariance, scale invariance, and inversion invariance. In addition, we performed experiments that retained other parameters from the experiments and added random modifications to the brightness, saturation, and contrast of the input images in the data augmentation to determine whether these factors have an impact on the performance of models. Additionally, we randomly clipped them to the size of 256 × 256 for the second task, which allows a larger batch size to improve the efficiency of training. During training, we selected one or more of the above strategies to dynamically augment the data. The data augmentation improves the robustness of the models effectively.

### Loss Calculation

We used the Cross-Entropy Loss $L_{ce}$ as the loss function for both two tasks. In the first task, it is defined as:


(1) $$\begin{array}{l} L_{ce1}=\frac{1}{N}\underset{i}{\sum }-\left[y_{i}\log\left(p_{i}\right)+\left(1-y_{i}\right)\log\left(1-p_{i}\right)\right], \end{array}$$

where *y*_*i*_ taken from 0 and 1 represents the two BRVO statuses and *p*_*i*_ represents the probability that the CFP is predicted to be with NPA by the classification model.

In the second task, it is defined as:


(2) $$\begin{array}{l} L_{ce2}=\frac{1}{N}\underset{x,y}{\sum }-\left[y_{x,y}\log\left(p_{x,y}\right)+\left(1-y_{x,y}\right)\log\left(1-p_{x,y}\right)\right], \end{array}$$

where *y*_*x,y*_ taken from 0 and 1 indicates whether the pixel behaves as NPA and *p*_*x,y*_ represents the probability according to the segmentation model.

### Training Model

In both tasks, we performed one 5-fold cross-validation test to train our models for performance evaluation and comparison. In the first task, 133 CFPs with NPA and 141CFPs without NPA were split into five parts separately. For every part, we randomly took 20% of CFPs with NPA and 20% of CFPs without NPA without repetition so that the proportion of positive and negative samples in the training set and verification set is equal. Furthermore, in the second task, 133 CFPs with NPA were split into five groups. In our experiments, 4 groups were used for training and 1 group was used for testing. Then, we executed this process 5 times in a loop until each group was used as the training and testing objects.

Through trial and error, we finally set the hyper parameters as shown in Table 1 for the first task and in Table 2 for the second task. To make the model converge, we adjusted the learning rate as the training went on. In the first task, we set the initial learning rate to 0.0001 and reduced it after every epoch. In the second task, we set the initial learning rate to 0.001 and linearly decreased it to 0.0005 in the last thousand groups of epochs. We used an Adam optimizer (24) to update the model parameters for efficient stochastic optimization with little memory requirement.

**Table 1:** Hyper-parameters of the first task.

**Hyper-parameters**	**Values**
Epoch	100
Batch Size	5
Iteration	47
Learning Rate	$10^{-4}\times \left(1-{\left(\frac{epoch_{now}}{500}\right)}^{0.9}\right)$
Optimizer	Adam

**Table 2:** Hyper-parameters of the second task.

**Hyper-parameters**	**Values**
Epoch	4000
Batch Size	10
Iteration	10
Learning Rate	$10^{-3}\times \min\left(1,1-\frac{2\times \left(epoch_{now}-3000\right)}{epoch_{total}}\right)$
Optimizer	Adam

### Evaluation of the DL Models

Table 3 illustrates the confusion matrix using binary classification. In the result, we figured out the number of true positive (TP), true negative (TN), false positive (FP), and false negative (FN). TP represents the number that was correctly classified as positive, FP represents the number that was incorrectly classified as positive, FN represents the number that was incorrectly classified as negative, and TN represents the number that was correctly classified as negative.

**Table 3:** Confusion matrix.

**Total population (P+N)**		**Predicted condition**	
		**Positive (PP)**	**Negative (PN)**
Actual Condition	Positive (P)	True positive (TP)	False negative (FN)
	Negative (N)	False positive (FP)	True negative (TN)

In the classification task, cases with the NPA needing prompt laser photocoagulation were defined as positive, and the other cases were defined as negative. In the segmentation task, we defined the pixels of the NPA region in CFPs as positive, and the other pixels as negative.

To increase the accuracy of the evaluation indicators, we did not include the black edge around the circular fundus image. In addition, we calculated the model accuracy, precision, recall rate, F1, receiver operating characteristic (ROC), and area under the curve (AUC).

Accuracy describes the proportion of correct predictions defined as:


(3) $$\begin{array}{l} Accuracy=\frac{TP+TN}{TP+TN+FP+FN} \end{array}$$

Precision (also called positive predictive value) describes the proportion of all predicted positive cases that are correctly predicted positive, which is defined as:


(4) $$\begin{array}{l} Precision=\frac{TP}{TP+FP} \end{array}$$

Recall or sensitivity (as it is called in psychology) describes the proportion of real positive cases that are correctly predicted positive, which is defined as:


(5) $$\begin{array}{l} Recall=\frac{TP}{TP+FN} \end{array}$$

In the segmentation task, our proportion of positive and negative samples approached 1:2. At this time, accuracy may yield misleading results. Thus, more attention needs to be paid to precision and recall (25).

F1 score was used to calculate the harmonic mean of the precision rate and the recall rate.


(6) $$\begin{array}{l} F1=\frac{2}{\frac{1}{precision}+\frac{1}{recall}} \end{array}$$

The ROC curve shows the FP rate on the *x*-axis and the true positive rate on the *y*-axis when the positive identification threshold is different. In addition, the AUC is the area under the value ROC curve. The higher the value of AUC, the better the performance of the model.

**Figure 4:**
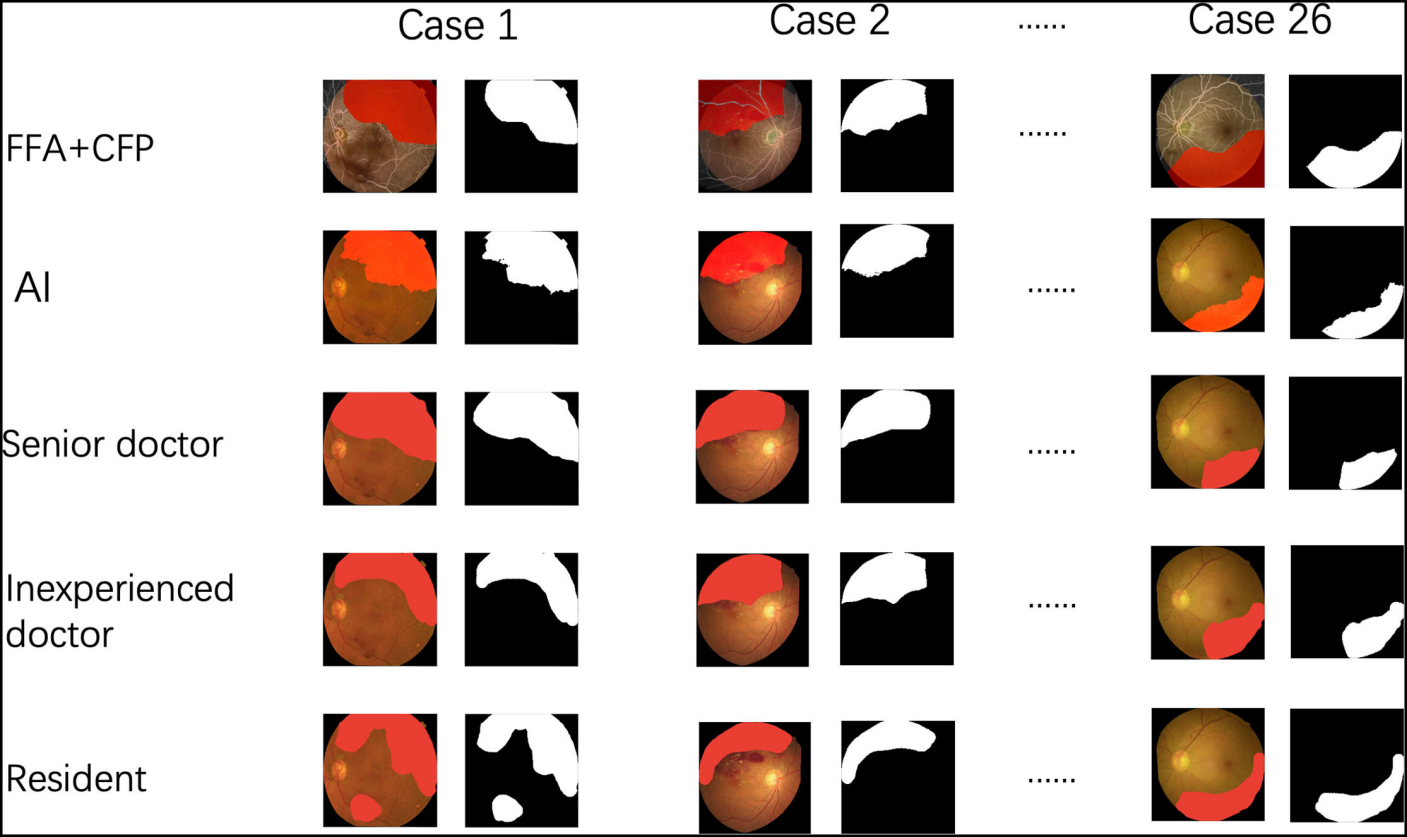
The procedure of the comparison among the artificial intelligence (AI) and three groups of ophthalmologists.

In addition, we compared the performance among the AI and three groups (number of each group = 3) of ophthalmologists with different experiences. The senior group had doctors with over 10 years of clinical training. The inexperienced group comprised doctors with <3 years of experience and the resident group had doctors with an experience of <1 year. All of the doctors were blind to all the cases and were guided to use the method to label the areas they considered to be NPA on the CFPs as mentioned above (Figure 4).

The TP, TN, FP, and FN of each CFP were averaged among three doctors and were compared with those in AI using an independent *T*-test. Data were expressed as mean ± standard deviation (SD). A *p-value* < 0.05 was considered statistically significant.

## Results

In total, 274 BRVO images from 274 patients (mean age: 66.3 ± 10.6 years; 141 men and 133 women; 139 left eyes and 135 right eyes) were analyzed.

In the first DL model, the recall, precision, accuracy, and AUC were 0.75 ± 0.08, 0.80 ± 0.07, 0.79 ± 0.02, and 0.82 ± 0.03, respectively, for predicting the necessity of laser photocoagulation for BRVO CFPs.

**Figure 5:**
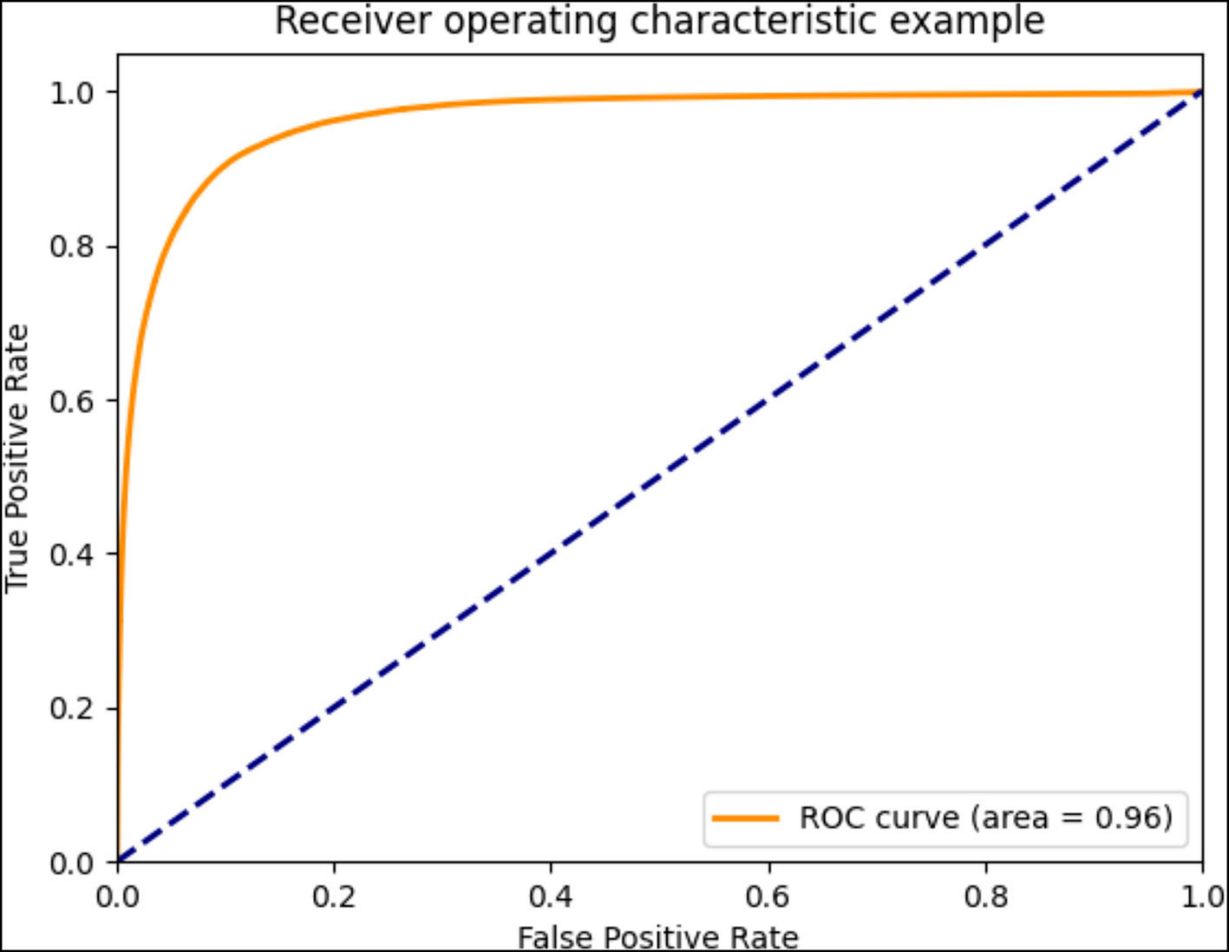
The receiver operating characteristic (ROC) curve of averaged 5-fold cross-validation.

The standard NPA was annotated by senior doctors based on FFA images (Figure 2). With regard to the second DL model for predicting NPA of BRVO, the recall was 0.74 ± 0.05, the precision was 0.87 ± 0.02, and the accuracy was 0.89 ± 0.02. The ROC of an average 5-fold cross validation is shown in Figure 5, and the value of AUC obtained was 0.96 ± 0.02 (Table 4). In addition, the prediction ability of models that data augmented with brightness, saturation, and contrast did not show a significant difference (Supplementary Table 1).

**Table 4:** Evaluation indicators obtained by a 5-fold cross-validation.

**ID**	**Accuracy**	**Precision**	**Recall**	**F1**	**AUC**
0	0.87	0.84	0.69	0.76	0.94
1	0.90	0.90	0.75	0.82	0.97
2	0.88	0.87	0.67	0.76	0.94
3	0.90	0.88	0.76	0.82	0.97
4	0.91	0.87	0.82	0.84	0.98
Average	0.89 ± 0.02	0.87 ± 0.02	0.74 ± 0.05	0.80 ± 0.03	0.96 ± 0.02

*AUC, area under the curve; F1, F1 score is to calculate the harmonic mean of the precision rate and the recall rate*.

In comparison with three independent doctors, our DL model was nearly comparable with the senior doctors in segmenting the NPA and was significantly better than the inexperienced doctors and residents (Table 5).

**Table 5:** Performance of DL model and senior doctor in identifying non-perfusion area (NPA).

	**Accuracy**	**Precision**	**Recall**	**F1**
**Senior doctor**	0.91 ± 0.04	0.79 ± 0.17	0.89 ± 0.07	0.82 ± 0.12
**DL model**	0.90 ± 0.05	0.87 ± 0.23	0.70 ± 0.24	0.76 ± 0.22
**t**	−0.21	1.77	−4.11	−1.50
**p**	0.83	0.09	0.00	0.15

*DL, deep learning; t, t statistic; p, p-value*.

## Discussion

In this study, the DL model identifying and segmenting the NPA from CFPs of eyes with BRVO showed high recall, precision, accuracy, and good performance of AUC. The DL model might facilitate the clinical decision for oculists in the treatment of BRVO with no need for invasive FFAs.

At present, with the rapid development of DL models, such as deep CNNs (26, 27), DL models have been intensively applied in ophthalmology (14), such as in detecting diabetic retinopathy (28), age-related macular degeneration (29), RVO (18, 30), and vitreous-retinal interface disorder (31) with CFPs or OCT images. Recently, studies have shown the application of AI for identifying microaneurysms (10, 32, 33) and NPA in DR based on FFA images (28, 34). However, FFA is an invasive method and is seldom used during surgery. As for the OCTA, a non-invasive method, it can identify the NPA (18) but with a relatively limited field. Here, our results show the possibility of detection of NPA using DL models with non-invasive CFPs. The results further demonstrated that the performance of the DL model may be close to an experienced ophthalmologist, which means that the algorithm can assist junior doctors to identify the NPA directly from the CFPs. DL models were compared with individual doctors, which has also been reported in previous studies (12, 13).

In some recent studies, DL has been reported to automatically identify NPA areas, especially in patients with diabetic retinopathy (DR) complicated by DME and retinal NV.

However, these identification models were almost achieved based on FFA images, or occasionally on wide-angle OCTA, which is still in their infancy, expensive, and not widely used. Although FFA is the gold standard to identify NPA, there are some limitations of FFA as described above. Notably, the FFA examination is invasive, and the quality of images may be influenced by eye movement, the poor focus of the camera, or uneven illumination due to the prolonged examination time. These studies also did not achieve automatic registration of FFA images and CFPs.

Our study creatively proposed to identify and segment NPAs only based on CFPs without the need for FFA and validate it for the first time in patients with BRVO. The performance reached by our DL models was similar to that obtained by previous models and human grader agreement. This convenient and inexpensive model can greatly aid those patients with contraindication of sodium fluorescein, as well as relieve the pressures of the exponential increase in clinical appointments. Our models can also facilitate ophthalmology clinicians, especially enabling junior doctors to make laser treatment decisions. The combination of the DL model and CFPs might also facilitate telemedicine. This approach may be particularly useful in areas with a shortage of FFA instruments or experienced examiners. With a clear CFP, patients with BRVO can immediately receive a precise segmentation of NPA and be guided with further laser photocoagulation (35). This study is an in-depth study from computer-aided diagnosis to treatment, and the potential use of this DL algorithm will be an outcome measure in clinical trials and a decision tool in clinical practice, which will be the theoretical basis for the application of intelligent guided laser (34, 36).

The present study also had some limitations. First, we compared only CFP images of BRVO eyes and did not include CFP images of normal eyes or other retinal diseases, which means that the FP possibility was not evaluated. Second, the scan area of CFPs was not large enough to detect the entire NPA in the montage FFA images, which calls for further training of peripheral CFPs or wide-field CFPs. Third, the sample size included was relatively small, and further studies using larger samples and involving other retinal diseases, such as DR, are required to evaluate the performance and versatility of DL for the detection of NPA.

In conclusion, our study proposed a conception of detecting the NPA directly from a CFP in the absence of FFA. Here, we first tried in the eyes with BRVO and demonstrated the DL model with a high level of accuracy. This model can also potentially be developed for the identification of NPA of the peripheral retina and other diseases, such as DR in the future.

## Data Availability Statement

The original contributions presented in the study are included in the article/Supplementary Material, further inquiries can be directed to the corresponding author/s.

## Ethics Statement

The study was conducted in accordance with the Declaration of Helsinki, and the study protocol was approved by the Ethics Committee of First Affiliated Hospital of Nanjing Medical University (2021-SR-330).

## Author Contributions

The research project was designed by ZH and QL and organized by JM, ZH, and QL. Data collection and labeling were performed by JM, WZ, NS, XW, ZP, and JH. DL modeled the design and training was performed by JY, KH, and QC. Statistical analysis was conducted by JM, JY, and YF. The test of the DL model was performed by YF, JH, PX, and SY. The first draft of the manuscript was written by JM and ZH, and the manuscript was reviewed and critiqued by ZH and QL. All authors contributed to the article and approved the submitted version.

## Funding

This study was supported by the National Natural Science Foundation of China (81900875 and 12027808 to ZH 81970821 and 81770973 to QL); the Key Research and Development Program of Jiangsu Province (BE2018131 to SY); and the Natural Science Foundation of Jiangsu Province (BK20191059 to ZH). The funders had no role in the study design, data collection, and analysis, decision to publish, or preparation of the manuscript.

## Conflict of Interest

The authors declare that the research was conducted in the absence of any commercial or financial relationships that could be construed as a potential conflict of interest.

## Publisher's Note

All claims expressed in this article are solely those of the authors and do not necessarily represent those of their affiliated organizations, or those of the publisher, the editors and the reviewers. Any product that may be evaluated in this article, or claim that may be made by its manufacturer, is not guaranteed or endorsed by the publisher.

## Footnotes

^1^VGG Net is the name of a convolutional neural network (CNN) invented by Simonyan and Zisserman from Visual Geometry Group (VGG) at University of Oxford in 2014.
