# Supplementary material for: Deep Learning Models for Segmenting Non-perfusion Area of Color Fundus Photographs in Patients With Branch Retinal Vein Occlusion
Source: Front Med (Lausanne). 2022 Jun 30;9:794045. doi: 10.3389/fmed.2022.794045 (PMC9279621; doi:10.3389/fmed.2022.794045)
Supplement: Supplementary file 1 [file Data_Sheet_1.docx]

| **Supplement Table 1. Comparison of Evaluation indicators** | | | | | |
| --- | --- | --- | --- | --- | --- |
|  | **Accuracy** | **Precision** | **Recall** | **F1** | **AUC** |
| **MODEL 1** |  |  |  |  |  |
| **Pre** | 0.79 ± 0.02 | 0.80 ± 0.07 | 0.75 ± 0.08 | 0.77 ± 0.04 | 0.82 ± 0.03 |
| **Post** | 0.78 ± 0.05 | 0.82 ± 0.12 | 0.72 ± 0.05 | 0.76 ± 0.05 | 0.81 ± 0.05 |
| **t** | 0.64 | -0.53 | 0.71 | 0.34 | 0.02 |
| **P** | 0.55 | 0.62 | 0.51 | 0.75 | 0.98 |
| **MODEL 2** |  |  |  |  |  |
| **Pre** | 0.89 ± 0.02 | 0.87 ± 0.02 | 0.74 ± 0.05 | 0.80 ± 0.03 | 0.96 ± 0.02 |
| **Post** | 0.89 ± 0.02 | 0.88 ± 0.01 | 0.73 ± 0.06 | 0.80 ± 0.04 | 0.96 ± 0.01 |
| **t** | -0.45 | -1.53 | 0.01 | -0.42 | -1.15 |
| **p** | 0.65 | 0.13 | 0.99 | 0.68 | 0.25 |
| **Pre**: original Evaluation indicators; **Post**: Evaluation indicators with changes of image brightness, saturation and contrast; **t**: t statistic; p: **p** value. | | | | | |

| **Supplement Table 2. Performance of DL model and doctors in identifying NPA** | | | |  |
| --- | --- | --- | --- | --- |
|  | **Accuracy** | **Precision** | **Recall** | **F1** |
| **Senior doctors** | 0.91±0.04 | 0.79±0.17 | 0.89±0.07 | 0.82±0.12 |
| **DL model** | 0.90±0.05 | 0.87±0.23 | 0.70±0.24 | 0.76±0.22 |
| **Inexperienced doctors** | 0.90±0.04 | 0.77±0.16 | 0.85±0.09 | 0.80±0.12 |
| **Residents** | 0.85±0.06 | 0.72±0.15 | 0.76±0.13 | 0.72±0.13 |
| **DL**: deep learning | | | |  |
